# Evaluation of the antimicrobial activity and compressive strength of a dental cement modified using plant extract mixture

**DOI:** 10.1007/s10856-020-06455-w

**Published:** 2020-11-28

**Authors:** Lamia Singer, Gabriele Bierbaum, Katja Kehl, Christoph Bourauel

**Affiliations:** 1 Oral Technology, University Hospital Bonn, Bonn, Germany; 2 Institute of Medical Microbiology, Immunology and Parasitology, University Hospital Bonn, Bonn, Germany; 3 Endowed Chair of Oral Technology, University Hospital Bonn, Bonn, Germany

## Abstract

Literature lacks sufficient data regarding addition of natural antibacterial agents to glass ionomer cement (GICs). Hence, the aim of the study was to increase the antimicrobial properties of GICs through its modification with mixture of plant extracts to be evaluated along with an 0.5% chlorohexidine-modified GIC (CHX-GIC) with regard to biological and compressive strength properties. Conventional GIC (freeze-dried version) and CHX were used. Alcoholic extract of *Salvadora persica, Olea europaea*, and *Ficus carcia* leaves were prepared using a Soxhlet extractor for 12 h. The plant extract mixture (PE) was added in three different proportions to the water used for preparation of the dental cement (Group 1:1 PE, 2:1 PE, and 1:2 PE). Specimens were then prepared and tested against the unmodified GIC (control) and the 0.5% CHX-GIC. Chemical analysis of the extract mixture was performed using Gas chromatography–mass spectrometry. Antimicrobial activity was evaluated using agar diffusion assay against *Micrococcus luteus* and *Streptoccocus mutans*. Compressive strength was evaluated according to ISO 9917-1:2007 using a Zwick testing machine at a crosshead speed of 0.5 mm/min. Antimicrobial activity against *Streptoccocus mutans* was significantly increased for all the extract-modified materials compared to the unmodified cement, and the highest concentration was comparable to the CHX-GIC mixture. The activity against *Micrococcus luteus* was also significantly increased, but only for the material with the highest extract concentration, and here the CHX-GIC group showed statistically the highest antimicrobial activity. Compressive strength results revealed that there was no statistically significant difference between the different mixtures and the control except for the highest tested concentration that showed the highest mean values. The plant extracts (PEs) enhanced the antimicrobial activity against *S. mutans* and also against *M. luteus* in the higher concentration while compressive strength was improved by addition of the PE at higher concentrations.

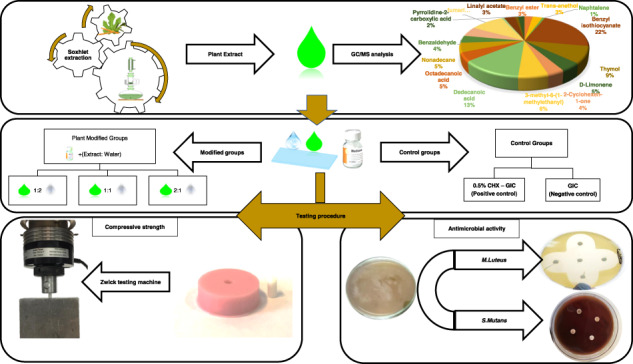

## Introduction

Glass ionomer cements (GICs) belong to a group of materials known as acid-base cements. The proper name for them, according to the International Organization for Standardization (ISO) is “glass polyalkenoate cement”, but the term “glass ionomer” is recognized as an acceptable trivial name, and is widely used within the dental community [1].

Over the past years, GICs have been the most commonly used water-based cements for final cementation of dental crowns, bridges, orthodontic brackets and atraumatic restorative treatment [2]. There are several properties that make glass ionomer a material of choice among which are their ability to bond adhesively to enamel and dentin, their biocompatibility and their ability to release fluoride ions over a prolonged period of time. Furthermore, GICs were shown to be rechargeable with fluoride ions [3, 4].

Literature has shown that microorganisms have been found to be viable for at least a period of 2 years under the GIC. Moreover, in spite of the fact that studies have shown that GICs release ~10 ppm of fluoride during the first 48 h following insertion into the cavity, this is still regarded low for achieving the desired antibacterial effects [5].

Therapeutic benefits may be gained by reinforcing GICs with additional antibacterial agents. Studies have been focusing on release or slow release of antibacterial agents such as antibiotics, zinc ions, silver ions, iodine, and most commonly chlorhexidine that is considered the gold standard for antibacterial applications. Several in vitro studies confirmed the enhancement of the biological properties of GIC when being incorporated with CHX [6–8].

Unfortunately, the incorporation of antibacterial agents in restorative materials frequently results in changes in the physical and mechanical properties of the restorative material over time, and might have short-term effectiveness and toxicity to surrounding tissues if the dose or release is not properly controlled. This is probably the reason why the combination of chlorhexidine and other antimicrobials with GICs is not yet employed in production [9, 10].

Many years ago, up to the advent of iatrochemistry in the 16th century, plants were used for treatment and prophylaxis in order to cure or alleviate illnesses. Phytomedicine can be defined as the herbal medicine that utilize different plant parts or extracts as a therapeutic or health-promoting agent. Herbal extracts showed an advantage of having beneficial effects without the risk of developing bacterial resistance [11, 12]. According to the World Health Organization, as many as 80% of the world’s people depend on traditional medicine (herbal) for their primary healthcare needs [13].

Among the effective medicinal plants are *Olea europaea, Ficus carcia*, and *Salvadora persica. Olea europaea (O. europaea)* is cultivated on a large scale in the Arabian Peninsula, India, and Asia, but the Mediterranean region is the main area of agricultural production [14, 15]. Recently, studies concerned with the medicinal properties of olive products have been focusing on olive polyphenols that have shown in animal and in vitro studies an antioxidant and an antimicrobial property [15].

*Ficus Carcia (F. carcia)* belongs to the mulberry tree family (Moraceae) which is one of the oldest harvested fruits in the world [16, 17]. Phytochemical studies on *F. carica* leaves revealed the presence of numerous bioactive compounds such as phenols, flavonoids, tannins, alkaloids, and saponins. Owing to these compounds *F. carcia* was reported to have antioxidant, anti-inflammatory, antiviral, and antibacterial activities [18, 19].

*Salvadora persica (S. persica)*, of the family Salvadoraceae, is an evergreen shrub, with a short trunk 4–6 m tall, smooth green leaves and white bark. *S. persica* has a wide geographic distribution ranging from India, Nepal in the east through Pakistan and Egypt in the west, and from Central Africa to Southwestern Africa [20]. Miswak, a chewing stick is prepared from *S. persica* roots or stems. It is used as an toothbrush stick for oral hygiene and in treatment of gum inflammation. It has been shown that an extract of miswak *(S. persica)* possesses different antimicrobial and antifungal properties due to the presence of trimethylamine, chlorides, fluoride, silica (Si02), sulfur, saponins, flavonoids, and phenols [20–22].

Literature lacks sufficient data regarding addition of natural antibacterial agents to GICs, despite that some of them have shown effective results against cariogenic salivary flora as mouthwashes or toothpaste. Hence, the aim of the study was to increase the antimicrobial properties of GICs through its modification by mixture of *S. persica, F. carcia* and *O. europaea* extracts to be evaluated along with an 0.5% CHX-modified GIC with regard to biological and compressive strength properties.

## Materials and methods

### Preparation of the three-plant extract mixture

*S. persica, F. carcia*, and *O. europaea* plants were used to prepare the extracts. Each of these plant parts was separately washed, dried, ground into powder, and added to the thimble of a Soxhlet extractor. Each extraction process was performed using ethyl alcohol (70%) alcohol for several hours. The extraction products from each plant was then filtered, proportioned to prepare a mixture of them all. The plant mixture was then placed at 37 °C in a rotary evaporator (Buchi Rotavapor R-300, Essen, Germany) to remove the ethanol leaving a crude mix that was stored in the fridge in a closed flask at 4 °C until use [10].

### Preparation of GIC, extract and CHX combinations and specimen grouping

Conventional, freeze-dried (powder/water) GIC, hand mix version (Medicem aqua, Promedica GmbH, Neumunster, Germany, Lot 1849261) was used.

The tested groups were prepared by either modifying the water used for preparation of GIC with different concentrations of the PE mixture or by adding CHX to the powder of GIC. These modified groups were then compared with a non-modified GIC as a control group (Table 1).

**Table 1:** Specimens’ grouping

Group name	Description
1—Control	Conventional, unmodified GIC.
Plant extract-modified groups (PE): 2—2:1 PE 3—1:2 PE 4—2:1 PE	The prepared plant extract (PE) was added to the water used for the preparation of GIC in three different extract to water mass ratios.
5—CHX-GIC	0.5% CHX diacetate (w/w) (Merck KGaA, Darmstadt, Germany) was added to the powder of GIC to be mixed with distilled water.

For the five groups, all specimens were mixed at a temperature of 23 ± 1 °C and a relative humidity of 50 ± 10% as per the powder/water ratio prescribed by the manufacturer (1:2). Freshly mixed specimens were prepared for each testing procedure.

### Chemical analysis of plant extract mixture (GC/MS)

The analysis was done at the Agriculture Research Center, Giza, Egypt using a gas chromatography (GC) (Agilent Technologies 7890A) interfaced with a mass-selective detector (MS) (MSD, Agilent 7000). One milliliter of the PE was diluted in diethylether and injected to analyse its chemical constituents. The GC was equipped with a polar Agilent HP-5ms (5%-phenyl methyl poly siloxane) and a capillary column (30 m, 0.25 mm inner diameter, and 0.25 µm film thickness). The injector and detector temperatures were set at 200 °C and 250 °C respectively. The carrier gas was helium and delivered at a linear velocity of 1 ml/min. Mass spectra were obtained at 70 eV ionization potential, acquisition mass range of 50–800 *m*/*z* in positive mode, and an interface temperature of 250 °C. The quantification of all identified components was investigated using a percent relative peak area. A tentative identification of the compounds was performed based on the comparison of their relative retention time and mass spectra with those of the of the authentic compounds and by computer matching with NIST and WILEY library as well as by comparison of the fragmentation pattern of the mass spectral data with those reported in literature [23].

### Agar well diffusion assay

Two gram-positive bacterial strains were used in the current study, *Streptococcus mutans* (DSMZ 20523) and *Micrococcus luteus* (DSMZ 4698).

MH agar plates were inoculated with suspensions of the indicator strains, adjusted to a 0.5 McFarland standard, equivalent to an *E. coli* suspension between 1 × 10^8^ and 2 × 10^8^ CFU/ml. After removing the suspension by pipetting, plates were dried for 20 min.

#### Specimens’ preparation for antimicrobial testing

The powder and liquid of GIC for each group were mixed with sterile spatulas according to the manufacturer instructions. Nine Petri dishes were used for each bacterial strain. Four wells (5 mm diameter) were prepared in each plate using a sterile cork borer so that each plate could receive the freshly mixed, unset control and modified groups with the different concentrations. Seven specimens were prepared for each group. For monitoring the antibacterial effect of the tested groups, the plates were incubated (Heraeus GmbH & Co. KG, Hanau, Germany) at 37 ± 1 °C for 48 h to allow the microorganisms to grow, and then the diameters of the circular inhibition zones around the samples were measured by using a digital micrometer [5, 24].

### Compressive strength

Compressive strength was evaluated according to ISO 9917-1:2007 using cylindrical molds (4.0 mm diameter × 6.0 mm height). Ten specimens were prepared for each group, powder and liquid were mixed according to the manufacturer’s instructions (1:2). Then materials were packed into the mold between polyester strips and thick glass plates on both sides to obtain a smooth surface. One hour later specimens were removed from the mold, grinded with silicon carbide paper and stored in deionised water for 24 h. Malformed specimens or those with voids were discarded. The diameter of each specimen was checked using a digital micrometer gauge (Digimatic, Mitutoyo Europe GmbH, Neuss, Germany). The specimens were then placed in vertical position in a Zwick universal testing machine (Zwick Zmart Pro, ZwickRoell GmbH & Co. KG, Ulm, Germany). Compressive load was applied on the long axis of the specimens at a crosshead speed of 0.5 mm/min until fracture. The maximum force applied when the specimen fractures was recorded to calculate the compressive strength values in MPa [25].

### Statistical analysis

All variables are numerical data presented as mean and standard deviation (SD). Normality test Shapiro–Wilk was used to examine whether or not the variables follow a normal distribution. All quantitative variables showed parametric distribution; therefore, One-way analysis of variance (ANOVA) was used for comparison between the groups. Tukey’s post hoc test was used for pairwise comparison between the groups when ANOVA test is significant. The significance level was set at *P* ≤ 0.05. Statistical analysis was performed using Minitab 17.1.0 for Microsoft Windows.

## Results

### Chemical analysis of plant extract mixture

Gas chromatography–mass spectrometry (GC/MS) revealed the presence of 38 volatile and semi-volatile compounds as summarized in (Table 2).

**Table 2:** Results of gas chromatography–mass spectrometry analysis

	Retention time (min)	Compounds	% Area
1	3.448	6,7-Dimethyl-4-hydroxycoumarin	4.78
2	3.907	6-Methylchromanone	0.87
3	4.199	3,4,5-Trimethoxycinnamic acid	0.86
4	4.572	o-Cymene	0.63
5	4.633	α-Pinene	11.01
6	5.092	7-Methoxy-3-(4-methoxyphenyl)coumarin	1.99
7	5.724	Limonene	2.53
8	6.232	Terpinolene	1.77
9	6.741	Myrtenol	2.46
10	7.163	p-Mentha-3,8-diene	1.63
11	7.364	α-Thujenal	3.73
12	8.184	Bornyl acetate	1.41
13	8.93	α-Terpineol	3.02
14	9.25	α-Selinene	4.34
15	9.406	δ-Guaiene	0.94
16	9.964	Humulene	1.18
17	10.099	Longifolene	4.2
18	10.23	γ-Gurjunene	1.76
19	10.956	*cis*-Sesquisabinene hydrate	0.99
20	11.161	Farnesol	1.45
21	11.342	Himbaccol	2.65
22	13.441	β-Santalol	2.28
23	13.667	Lanceol, *cis*	4.65
24	13.888	α-Terpinyl acetate	2.77
25	13.966	3,6,3′,4′-Tetrahydroxyflavone	2.01
26	14.512	Kaur-16-ene	1.41
27	14.815	Squalene	4.33
28	14.922	Ledol	15.51
29	15.11	7,3′,4′,5′-Tetramethoxyflavanone	2.22
30	15.398	Quercetin 3′-methyl ether	2
31	16.566	p-Cresol, 2,2′-methylenebis(4-methyl-6-tert-butylphenol)	1.76
32	17.657	Apigenin 8-C-glucoside	0.65
33	18.006	2′-Hydroxy-2,4,4′,5-tetramethoxychalcone	0.9
34	18.309	Juniperol	1.29
35	18.752	Isovitexin	0.17
36	19.814	6,2′,3′-Trimethoxyflavone	1.39
37	22.546	7-Hydroxychromanone	0.94
38	22.878	4-Hydroxy-7-methoxy-3-(4-methoxyphenyl) coumarin	1.56

### Agar well diffusion assay for antimicrobial activity

#### Antimicrobial activity against *S. mutans*

**Fig. 1:**
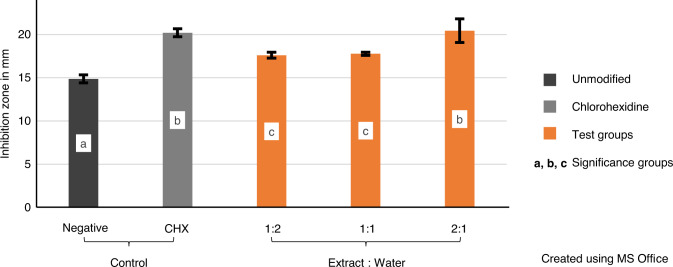
Mean values of inhibition zones (mm) showing intergroup comparison against *Streptococcus mutans* and 95% confidence interval of tested groups. Groups that do not share a letter are significantly different

The variables showed parametric distribution and thus one-way ANOVA was used to test the antibacterial effect of the plants’ extract against *S. mutans* followed by Tukey’s post hoc for pairwise comparison between the tested groups (Fig. 1). An ANOVA indicated that there was a statistically significant antibacterial effect of the extract against *S. mutans*, *F* (4, 30) = 63.23, *P* value < 0001.

Post hoc comparison using Tukey’s test indicated that the mean values of the groups CHX-GIC and 2:1 PE (20.2, 20.4 respectively) were statistically significantly higher than the mean values of the remaining groups. Furthermore, there was statistically insignificant difference between the mean values of the groups 1:2 PE (17.6) and 1:1 PE (17.7) though they were statistically significantly higher than the mean of the control group (14.8).

#### Antimicrobial activity against *M. luteus*

**Fig. 2:**
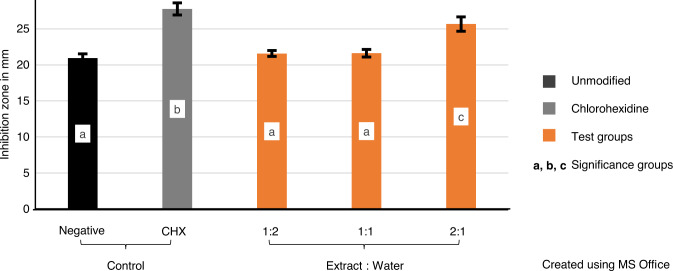
Mean values of inhibition zones (mm) showing intergroup comparison against *Micrococcus luteus* and 95% confidence interval of tested groups. Groups that do not share a letter are significantly different

The variables showed parametric distribution and thus one-way ANOVA was used to test the antibacterial effect of the plants’ extract against *M. luteus* followed by Tukey’s post hoc test for pairwise comparison between the tested groups (Fig. 2). An ANOVA indicated that there was statistically significant antibacterial effect of the extract against *M. luteus*, *F* (4, 30) = 109.87, *P* value < 0001.

Post hoc comparisons using Tukey’s test indicated that the mean value of the group CHX-GIC (27.7) is statistically significantly the highest among the tested groups, followed by the mean value of the group 2:1 PE (25.6) which was statistically significantly higher than the mean values of the groups control, 1:2 PE and 1:1 PE (20.9, 21.5, and 21.6 respectively).

### Compressive strength

**Fig. 3:**
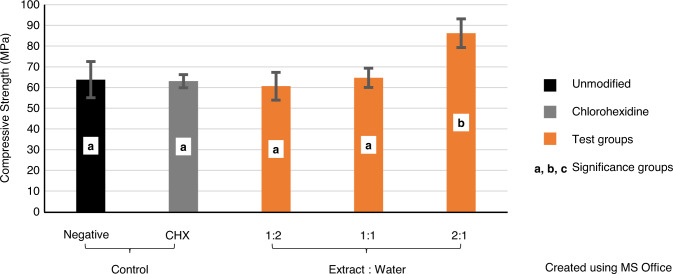
Mean compressive strength values (MPa) showing intergroup comparison and 95% confidence interval of tested groups. Groups that do not share a letter are significantly different

The variables showed parametric distribution and thus one-way ANOVA was used to test the compressive strength of the plants’ extract followed by Tukey’s post hoc test for pairwise comparison between the tested groups (Fig. 3). An ANOVA indicated that there was a statistically significant effect on the compressive strength, *F* (4, 45) = 13.94, *P* value < 0001.

Post hoc comparisons using Tukey’s test indicated that the mean value of the compressive strength of the group 2:1 PE (86.2) was statistically significantly higher than the mean values of all tested groups. Moreover, there was statistically insignificant difference between the mean values of the groups control, CHX-GIC, 1:2 PE and 1:1 PE (63.8, 63, 60.6, and 64.6 respectively).

## Discussion

Numerous studies revealed that incorporation of antibacterial agents in restorative materials has many therapeutic benefits, but frequently results in impaired physical and mechanical properties. These limitations provoke the need to develop some innovative strategies that can act against dental caries without altering the material properties. One of such strategies is to explore the abundantly available medicinal plants in nature that have proven a profound antimicrobial activity [8, 26].

In the present study, *Salvadora persica, Ficus carcia*, and *Olea europaea* were extracted using alcohol to prepare extract mixtures of the three plants. This extract mixture was used to modify a conventional freeze-dried GIC by adding it to the water used for the final mixture at three different volume ratios of extract to water (1:1 PE, 1:2 PE, 2:1 PE). The extract-modified materials were evaluated and compared with a conventional GIC (Control) and 0.5% CHX-modified GIC (CHX-GIC) with regard to the antimicrobial and compressive strength properties.

Chlorhexidine is a broadband antimicrobial agent with a bactericidal and bacteriostatic effect [27]. The antibacterial effect of CHX is concentration dependent, whereas high concentrations of CHX might negatively affect the physical and mechanical properties of GIC [28, 29]. 0.5% of CHX (w/w) was chosen in the current study to be added to GIC powder, based on studies reporting that this percentage might be the best option for incorporation of CHX into GIC, since antibacterial activity increased and the physical–mechanical properties were not compromised [30–32].

Antimicrobial activity was assessed using the agar well diffusion method against *S. mutans* and *M. luteus*. These microorganisms were chosen because *S. mutans* has been identified as the major causative agent of dental caries, playing a main role in carbohydrate fermentation and acid production [33], whereas M. *luteus* is a very sensitive indicator strain for the release of antibacterial compounds. *M. luteus* was also the most predominant opportunistic pathogen among all isolates from the teeth and gums of children belonging to age group 7–16 years [34].

The agar disc diffusion method, developed in 1940, is one of the most common methods used in many laboratories for routine antimicrobial susceptibility testing. Although not all fastidious bacteria can be tested accurately by this method, standardization has been made to test bacteria like *M. luteus* or Haemophilus influenzae using specific culture media and incubation parameters. Nevertheless, disk-diffusion assay offers many advantages over other methods including simplicity, low cost, the ability to test enormous numbers of microorganisms and antimicrobial agents [35].

Results of agar diffusion assays against *S. mutans* showed that PEs with the different concentrations had a significant effect on inhibition of bacterial growth compared to the control group. Such an effect was more pronounced with increasing the concentration of the extract as in group 2:1 PE that showed the statistically highest inhibition zones compared to groups 1:1 PE and 1:2 PE; see Fig. 1.

These results are in accordance with Ribeiro and Erickson [36] and Botelho [37], who reported that the antimicrobial activity was dependent upon the concentration of the disinfectant added. However, the results contradict Jedrychowski et al. [28] who indicated a no effects of dose response. Moreover, the 2:1 PE cement showed comparable results to CHX-GIC cement with both being significantly the highest among all other tested mixtures.

For *M. luteus*, mean inhibition zones of 2:1 PE plant modified group were significantly higher than the control group. However, CHX-GIC group was significantly the highest followed by 2:1 PE group compared to the other tested groups, see Fig. 2. This can be explained on the basis that *M. luteus* is very sensitive to chlorhexidine which can be efficiently taken up by the bacteria according to Wendel et al. [38].

The antimicrobial activity might be attributed to the different phytochemical constituents in each of the three incorporated PE. Identification of volatile and semi-volatile compounds in the PE in the current study was made using a combination of two analytical techniques; GC and mass spectrometry. GC can separate compounds with high resolution, but it cannot identify them. Mass spectrometry can provide detailed structural information on most compounds to be accurately identified and quantified after their separation [23].

GC/MS analysis of the extract mixture revealed the presence of 38 compounds including; monoterpenes hydrocarbons (α-pinene, limonene, 0-cymene), monoterpene alcohols (linalool, α-terpineol), and sesquiterpene (Himbaccol, α-Selinene, ledol, Juniperol). All of these terpenoids are thought to cause membrane disruption that is triggered by the lipophilic compounds [39].

Coumarins (6,7-Dimethyl-4-hydroxycoumarin, 7-Methoxy-3-(4-methoxyphenyl) coumarin, 2′-Hydroxy-2,4,4′,5-tetramethoxychalcone coumarin,), Trimethoxycinnamic acid and Phenols (7-Hydroxychromanone and p-Cresol) were also identified. Studies have reported that coumarins as well as phenols exhibited strong antibacterial activity against both Gram-positive and Gram-negative strains by damaging the bacterial cell membrane causing denaturation of protein and affecting cell membrane permeability [40, 41].

Moreover, flavonoids and saponin have been detected at various percentages in the PE. Flavonoids (Isovitexin, 2′-Hydroxy-2,4,4′,5-tetramethoxychalcone, Quercetin 3′-methyl ether and 7,3′,4′,5′-Tetramethoxyflavanone, Apigenin 8-C-glucoside) antimicrobial efficacy is due to inhibition of nucleic acid synthesis and alteration of cytoplasmic membrane function [42]. Whereas, saponin (Squalene, Kaur-16-ene) causes leakage of proteins and certain enzymes from the bacterial cells [43].

Chlorohexidine’s mechanism of action was explained by the release of positively charged cationic molecules through the dissociation of CHX salt. These cationic molecules bind to the negatively charged bacterial cell walls where, at low concentrations, the result is bacteriostatic while at high concentrations, membrane disruption occurs resulting in cell death [44].

The clinical success of a material is defined by its ability to withstand the stresses and strains induced during mastication and function. The most commonly used strength value to characterize dental cements is the compressive strength. The minimum compressive strength required according to ISO 9917 (2007) is 50 MPa for base/lining and 100 MPa for restorations. Therefore, it was important to evaluate the compressive strength when modifying the GIC [25].

Compressive strength test was performed after 24 h storage as it is recommended to compare the mechanical properties of GIC between periods of 1 and 24 h or more because their final setting is achieved after 24 h, and they usually present lower strength values during the first hours [45].

Results of compressive strength tests showed insignificant difference between all of the control, 1:1 PE, 1:2 PE, and CHX-GIC groups; see Fig. 3. Such findings are in accordance with Farret et al. [46], Marti et al. [47], and Jaidka et al. [32], who stated that incorporation of antimicrobial agents at certain concentrations did not affect the compressive strength properties of GIC.

However, the current study based on its conditions and findings contradicts with Cefaly et al. [48], Ewoldsen et al. [49], and Sanders et al. [50]. The reduction in compressive strength with antimicrobials in the former studies was attributed to the alteration of the powder/liquid ratio of the mixture and/or interference of antimicrobials with the cross-linking of GIC that occurs by the coordination of Al^3+^ and Ca^2+^with the COOH groups on the acidic polymers, thus decreasing the mechanical properties. Moreover, the majority of antimicrobial agents are added in the form of powders that easily absorb water, decreasing the compressive strength of the GIC. This explanation does not comply with the present study as the PE was added in the form of liquid and CHX powder was added in a very small percentage that did not seem to cause such a problem [49, 50].

Surprisingly, the 2:1 PE group showed a significant improvement in the compressive strength values from the control and the other modified groups; see Fig. 3. This could be explained on the basis of the phytochemical analysis of PEs that revealed the presence of silica in *Salvadora persica* [51]. Lihua et al. [52] and Tjandrawinata et al. [53] proved that addition of silica fillers improves the compressive strength of conventional GIC through the ability of silica to adhere to the matrix by chemical bonding and hence reinforcing the GIC.

Moreover, Cinnamic and bornyl acetic carboxylic acids were identified by GC/MS in the extract. It was assumed that by adding these acids, to glass ionomer liquids, the degree of cross-linking increases together with polysalt bridge formation and subsequently the mechanical properties of the set cement. This was in accordance with Prentice et al. [54], who showed that increasing the concentration of polyacrylic acid with another carboxylic acid considerably reduced the pH, which improves the release of ions from the surface of the glass ionomer powder and increases the rate of cross-linking.

Further studies with respect to other bacterial strains, shear bond strength, and applicability in dental practice are in progress.

## Conclusion

- PEs enhanced the antimicrobial activity of GIC against *Streptococcus mutans,* while their effect against *Micrococcus luteus* was only pronounced at high extract concentrations.
- The compressive strength of GIC was improved by the addition of high concentration of PEs.

## References

[1] Sidhu S, Nicholson J (2016). A review of glass-ionomer cements for clinical dentistry. J Funct Biomater.

[2] Craig RG, Powers JM. Restorative dental materials. 11th ed. St. louis: Mosby-Elsevier Science Ltd; 2002.

[3] Nomot R, Komoriyama M, McCabe J, Hirano S (2004). Effect of mixing method on the porosity of encapsulated glass ionomer cement. Dent Mater.

[4] Wiegand A, Buchall W, Atti A (2007). Review on fluoride-releasing restorative materials—fluoride release and uptake characteristics, antibacterial activity and influence on caries formation. Dent Mater.

[5] Takahashi Y, Imazato S, Kaneshiro AV, Ebisu S, Frencken JE, Tay FR (2006). Antibacterial effects and physical properties of glass-ionomer cements containing chlorhexidine for the ART approach. Dent Mater.

[6] Palmer G, Jones F, Billington RW, Pearson G (2004). Chlorhexidine release from an experimental glass ionomer cement. Biomaterials..

[7] Boyd D, Li H, Tanner DA, Towler MR, Wall JG (2006). The antibacterial effects of zinc ion migration from zinc-based glass polyalkenoate cements. J Mater Sci Mater Med.

[8] Pinheiro SL, Simionato MR, Imparato JC, Oda M (2005). Antibacterial activity of glass-ionomer cement containing antibiotics on caries lesion microorganisms. Am J Dent.

[9] Yesilyurt K, Tasdemir T, Buruk K, Celik D (2009). Antibacterial activity and physical properties of glass-ionomer cements containing antibiotics. Oper Dent.

[10] El-Tatari A, de Soet J, de Gee J, Shelib A, van Amerongen E (2011). Influence of salvadora persica (miswak) extract on physical and antimicrobial properties of glass ionomer cement. Eur Arch Paediatr Dent.

[11] Jamshidi-Kia F, Lorigooini Z, Amini-Khoei H (2018). Medicinal plants: past history and future perspective. J Herbmed Pharmacol.

[12] Kelly K (2009). The history of medicine.

[13] Kumar G, Jalaluddin M, Rout P, Mohanty R, Dileep C (2013). Emerging trends of herbal care in dentistry. J Clin Diagn Res.

[14] Ryan D, Robards K (1998). Phenolic compounds in olives. Analyst..

[15] Vogel P, Kasper Machado I, Garavaglia J, Zani T, de Souza D, Morelo Dal Bosco S (2014). Polyphenols benefits of olive leaf (Olea europaea L*)* to human health. Nutr Hosp.

[16] Nirwana I, Rianti D, Soekartono H, Listyorini R, Basuki P (2018). Antibacterial activity of fig leaf (Ficus carica Linn.) extract against Enterococcus faecalis and its cytotoxicity effects on fibroblast cells. Vet World.

[17] Mawa S, Husain K, Jantan I (2013). Ficus carica L. (Moraceae): phytochemistry, traditional uses and biological activities. Evid Based Complement Altern Med.

[18] Veberic R, Colaric M, Stampar F (2008). Phenolic acids and flavonoids of fig fruit (Ficus carica L.) in the northern Mediterranean region. Food Chem.

[19] Solomon A, Golubowicz S, Yablowicz Z (2006). Antioxidant activities and anthocyanin content of fresh fruits of common fig *(Ficus carica L*.). J Agric Food Chem.

[20] Dahiya P, Kamal R, Luthra RP, Mishra R, Saini G (2012). Miswak: a periodontist’s perspective. J Ayurveda Integr Med.

[21] Patel PV, Shruthi S, Kumar S (2012). Clinical effect of miswak as an adjunct to tooth brushing on gingivitis. J Indian Soc Periodontol..

[22] Al-Sadhan, Almas K (1999). Miswak (chewing stick): a cultural and scientific heritage. Saudi Dent J.

[23] Santana P, Miranda M, Payrol J, Silva M, Hernández V, Peralta E (2013). Gas chromatography-mass spectrometry study from the leaves fractions obtained of vernonanthura patens (Kunth) H. Rob. Int J Org Chem.

[24] Mittal S, Soni H, Sharma DK, Mittal K, Pathania V, Sharma S (2015). Comparative evaluation of the antibacterial and physical properties of conventional glass ionomer cement containing chlorhexidine and antibiotics. J Int Soc Prev Community Dent.

[25] International Organization of Standardization. ISO 9917-1:2007-Dental-water-based cements. Part 1: Powder/liquid acid-base cements. Geneva: ISO; 2016.

[26] Botelho MG (2004). Compressive strength of glass ionomer cements with dental antibacterial agents. SADJ..

[27] Varoni E, Tarce M, Lodi G, Carrassi A (2012). Chlorhexidine (CHX) in dentistry: state of the art. Minerva Stomatol.

[28] Jedrychowski JR, Caputo AA, Kerper S (1983). Antibacterial and mechanical properties of restorative materials combined with chlorhexidines. J Oral Rehabil.

[29] Hoszek A, Ericson D (2008). In vitro fluoride release and the antibacterial effect of glass ionomers containing chlorhexidine gluconate. Oper Dent.

[30] Marti LM, Mata MD, Ferraz-Santos B, Azevedo ER, Giro EM, Zuanon AC (2014). Addition of chlorhexidine gluconate to a glass ionomer cement: a study on mechanical, physical and antibacterial properties. Braz Dent J..

[31] Becci O (2014). Influence of the addition of chlorhexidine diacetate on bond strength of a high-viscosity glass ionomer cement to sound and artificial caries-affected dentin. Rev Odontol.

[32] Jaidka S, Somani R, Singh DJ, Shafat S (2016). Comparative evaluation of compressive strength, diametral tensile strength and shear bond strength of GIC type IX, chlorhexidine-incorporated GIC and triclosan-incorporated GIC: an in vitro study. J Int Soc Prev Community Dent.

[33] Gross EL, Beall CJ, Kutsch SR, Firestone ND, Leys EJ, Griffen AL (2012). Beyond streptococcus mutans: dental caries onset linked to multiple species by 16S rRNA community analysis. PLoS ONE.

[34] Raju KS, Anitha L (2014). Isolation and identification of oral flora from individuals belonging to ages 7 to 16 years. TIJ’s Res J Sci IT Manag.

[35] Anitha Rani A, Jeeva SS, Punitha M, Packia Lexshmi J, Raja, Brindha J (2016). Biochemical and molecular analysis of bacteria isolated from dental caries. J Chem Pharm Res..

[36] Ribeiro J, Ericson D (1991). In vitro antibacterial effect of chlorhexidine added to glass-ionomer cements. Scand J Dent Res.

[37] Botelho MG (2003). Inhibitory effects on selected oral bacteria of antibacterial agents incorporated in a glass ionomer cement. Caries Res.

[38] Wendel SO, Menon S, Alshetaiwi H (2015). Cell based drug delivery: micrococcus luteus loaded neutrophils as chlorhexidine delivery vehicles in a mouse model of liver abscesses in cattle. PLoS ONE.

[39] Silva A, Rivas da Silva A, Lopes P, Barros de Azevedo M, Costa D, Alviano C (2012). Biological activities of α-pinene and β-pinene enantiomers. Molecules..

[40] Leal M, Ferreira G, Bezerra A, Matos A, Viana B (2000). Anticonceptive, anti-inflammatory and bronchodilator activities of Brazilian medicinal plants containing coumarin: a comparative study. J Ethnopharmacol.

[41] Działo M, Mierziak J, Korzun U, Preisner M, Szopa J, Kulma A (2016). The potential of plant phenolics in prevention and therapy of skin disorders. Int J Mol Sci.

[42] Liu Y, McKeever CL, Malik NSA (2017). Assessment of the antimicrobial activity of olive leaf extract against foodborne bacterial pathogens. Front Microbiol..

[43] Sun X, Yang X, Xue P, Zhang Z, Ren G (2019). Improved antibacterial effects of alkali-transformed saponin from quinoa husks against halitosis-related bacteria. BMC Complement Altern Med.

[44] Jones CG (1997). Chlorhexidine: is it still the gold standard?. Periodontol.

[45] Prosser HJ, Powis DR, Brant P, Wilson AD (1984). Characterization of glass-ionomer cements. The physical properties of current materials. J Dent.

[46] Farret MM, Lima EM, Mota EG, Hugo MS, Oshima HMS, Barth V (2011). Can we add chlorhexidine into glass ionomer cements for band cementation?. Angle Orthod.

[47] Marti M, Rizzato A, da Giro M, Zuanon E, Cilense A (2014). Effect of chlorhexidine gluconate on porosity and compressive strength of a glass ionomer cement. Rev de Odontologia UNESP.

[48] Cefaly G, Franco B, Mondelli L, Francisconi S, Fidela N (2003). Diametral tensile strength and water sorption of glass-ionomer cements used in atraumatic restorative treatment. J Appl Oral Sci.

[49] Ewoldsen N, Covey D, Lavin M (1997). The physical and adhesive properties of dental cements used for atraumatic restorative treatment. Spec Care Dent.

[50] Sanders BJ, Gregory RL, Moore K, Avery DR (2002). Antibacterial and physical properties of resin modified glass-ionomers combined with chlorhexidine. J Oral Rehabil.

[51] Haque MM, Alsareii SA (2015). A review of the therapeutic effects of using miswak (Salvadora Persica) on oral health. Saudi Med J.

[52] Lihua E, Irie M, Nagaoka N, Yamashiro T, Suzuki K (2010). Mechanical properties of a resin-modified glass ionomer cement for luting: effect of adding spherical silica filler. Dent Mater J.

[53] Tjandrawinata R, Irie M, Yoshida Y, Suzuki K (2004). Effect of adding spherical silica filler on physico-mechanical properties of resin modified glass-ionomer cement. Dent Mater J.

[54] Prentice L, Tyas M, Burrow M (2006). The effect of oxalic acid incorporation on the setting time and strength of a glass-ionomer cement. Acta Biomater.

